# Delayed neurological deficit due to a medially misplaced thoracic pedicle screw during adolescent idiopathic scoliosis correction: a complication 6 years in the making

**DOI:** 10.1007/s43390-024-00951-7

**Published:** 2024-09-04

**Authors:** Sudhir Suggala, Garrett A. Dyess, Olivier Darbin, Richard P. Menger

**Affiliations:** Department of Neurosurgery, University of South Alabama, 1601 Center Street, Suite 2D, Mobile, AL 36604 USA

**Keywords:** Delayed neurological deficit, Motor vehicle collision [MVC], Pedicle screw misplacement, Spine safety, Spine trauma

## Abstract

**Purpose:**

Neurological deficits developing years after pedicle screw misplacement is a rare phenomenon. Here, we report level IV evidence of a previously asymptomatic medial thoracic pedicle screw resulting in paraparesis after a motor vehicle accident.

**Methods:**

A 21-year-old male presented with acute onset of paraparesis following a motor vehicle collision. Six years prior this incident, the patient underwent a thoracolumbar fusion T4-L4 for AIS performed by an outside orthopedic surgeon. CT scan and CT myelogram illustrated decreased spinal canal diameter and cord compression from a medial T8 pedicle screw.

**Results:**

Surgical removal of the misplaced pedicle screw resulted in a gradual complete recovery sustained over a period of 2 years. This case is compared to those reported in the literature review between 1981 and 2019 concerning delayed neurological deterioration related to misplaced pedicle screw.

**Conclusion:**

This case reports a delayed neurological deficit implicating a misplaced pedicle screw. This phenomenon remains rare since 5 cases were reported in the literature over the last 4 decades. It calls into focus the need for confirmation of safe instrumentation during the intraoperative period. It also illustrates the potential difficult decision-making in regard to asymptomatic misplaced instrumentation.

**Level of evidence:**

IV

## Introduction

Pedicle screw fixation has revolutionized the spine deformity surgery and is now considered a standard of care for deformity correction. The technique of internal fixation with screws was first described by King [1948] and Boucher [1959] [1, 2]. Pedicle screw usage in thoracic spine was ushered by Suk, Lenke and Kim et al. [3, 4] in the contemporary era. Pedicle screws have allowed for superior coronal curve correction with lower pseudoarthrosis and implant failure rates as compared to other constructs such as hooks or wires. This is because of the 3-column fixation, greater spine derotation and robust technological development. A potential complication with this technique is the misplacement of the pedicle screw. Screw misplacement has an overall incidence of 0–42% despite technical advances [5]. In comparison, the rate of new neurological deficits in spinal deformity surgery ranges from 0.1– 3% [6]. The cause underlying these deficits include nerve root or spinal cord involvement and result from mechanisms like screw placement, direct trauma, bony or soft tissue compression during the deformity correction, and vascular ischemia. Risk factors for neurological injuries in scoliosis include congenital and neuromuscular scoliosis, hyper-kyphosis > 40°, Cobb’s angle > 90°, intraoperative osteotomies, increased BMI, blood loss and operative time. Neurologic deficits can occur intraoperatively or occur several hours or days after surgery [7]. Various adjunct technologies have been suggested [CT navigation, multimodal neuromonitoring, or robotics] to improve the accuracy of screw placement and detect complications at an early correctable stage. Another area of uncertainty is the return to activity time after a spine deformity surgery, especially in the presence of pedicle instrumentation. Among multiple recommendations, the recent AO spine guidelines give a good perspective [8].

In contrast, very late neurological deficits, without instrumentation failure, which occur months to years after spine deformity surgery are only sporadically reported in the literature. Here we present a case of delayed neurological deficit that occurred 6 years after a surgical correction of a 49° main thoracic curve. A now 21-year-old male presented with paraparesis following a motor vehicle high-speed collision. This case report is discussed and compared to the very few similar cases previously reported in the literature.

## Materials and methods

This is an IRB-approved, retrospective review of this patients’ charts, radiographs, and postoperative CT scans, who underwent posterior spinal fusion (PSF) revision with removal of pedicle screws in January 2021. Patient demographics such as sex, height and age were obtained from the charts. Cobb angle, kyphosis angles and fixation points from X-rays. Postoperative CT scan, radiographs and CT myelogram were analyzed by a radiologist to determine screw placements, compression of cord and percentage of spinal canal encroachment at the index level.

We conducted a literature review in the PubMed/MEDLINE search engine, in addition to Medical Subject Headings (MeSH) terms. “Scoliosis”, “AIS”, “Neurological deterioration”, “Delayed deficit”, “Late deterioration”, “Medial screw misplacement/malposition”, “Thoracic pedicle” were the MeSH terms used. Our review considered the first report published to the last report published (1981–2019). The language of publication was limited to English. We chose to include cases which developed neurological deficits and deteriorated at least 3 months after the first surgery; thus, completely excluding the immediate and early post-operative causes. Finally, 22 case reports were selected (Table 1).

**Table 1:** Prior research on delayed neurological deficit after the first deformity surgery

First author	Year of publication	Cases	Patient age/sex at presentation of deficit	Original surgery indication	Original surgery	Time to presentation	Presentation	Management	Cause of compression	Outcome
1. Eismont et al. [28]	1981	1	17Y/M	T4-L1 AIS	Harrington rod/hook correction, T4-L1 with fusion	2 years	Spastic paraparesis	T9-L2 left posterolateral decompression	Left T9-T11 bony hypertrophy	Complete recovery
2. Court-Brown et al. [29]	1982	1	59Y/F	T12-L4 idiopathic scoliosis	Harrington rod distraction and T12 to sacrum fusion	4 years	Spastic paraparesis	T12-L1 laminectomy and decompression, fixation and onlay fusion	Harrington rod fracture with T12-L1 pseudoarthrosis with bony mass compressing the dura bilaterally	Complete recovery
3. Bernard Jr et al. [30]	1983	2	1. 13Y/F 2. 12Y/M	1. T7-L2 thoracic kyphosis 2. T5-T11 right thoracic scoliosis, T11-L4 left lumbar scoliosis	1. T6-L4 PSF and T11 to L2 anterior release and interbody fusion with 18G sublaminar wires and rods 2. T4-L4 PSF and partial correction	1. 1.3 years 2. 1.6 years	1. Pain and progressive kyphosis 2. Left lumbar radiculopathy with L4, L5 hypoesthesia after fall	1. Removal of broken wires and rods and extension of proximal fusion to T2 2. Removal of broken wires and removal of pseudoarthrosis causing compression	1. Wire breakage and pseudoarthrosis 2. Wire breakage and pseudoarthrosis	1. Complete recovery 2. Complete recovery
4. Roy et al. [31]	1984	1	29Y/M	AIS	T5-sacrum fusion with Harrington rods and hooks	15 years	Paraplegia	Removal of hook and rod with laminectomy T4-T6	Cord compression due to broken rod and hook	No recovery
5. Kornberg et al. [32]	1985	1	42Y/F	Scoliosis	T2-L4 double Harrington rod	25 years	Left sciatica	L4-L5 laminectomy	Left lumbar hook with bony growth around it causing compression	Complete recovery
6. Hales et al. [33]	1989	4	1. 16Y/F 2. 36Y/F 3. 56Y/F 4. 60Y/F	1. T6-T12 right, L1-L4 left AIS 2. T10-L3 left TL curve 3. T7-T11 right, T2-L5 left 4. T5-T12 right, T11-L4 left	1. T5-L5 Harrington double rod with hooks 2. T10-L5 fixation Harrington rod with hooks 3. T11-L5 Harrington rod and hook 4. T10-L5 Harrington rod, hook, wires	1. 3 years 2. 2.4 years 3. 1.8 years 4. 8 months	1.Right sciatica 2.Right sciatica 3.Left sciatica 4.Left sciatica	1.Hardware removal 2.Hardware removal 3.Conservative 4.Hardware removal	1.Caudad hook migration at L5 with chronic Dural Tear 2.Hook, chronic dural tear 3.Hook migration at L5 4.Hook migration	1.Complete recovery 2.Incomplete recovery 3. Died due to other cause 4.Complete recovery
7. Savini et al. [34]	1990	3	No details available	Deformity surgery	Posterior spinal fusion for deformity	No details available	Spastic paraparesis	1, 2: Partial deformity correction and combined fusion (anterior and posterior) 3: Posterior spinal cord decompression and stabilization by combined fusion	1,2: Stretching of the spinal cord due to progression of deformity in kyphosis 3: Spinal cord compression due to bone overgrowth at the site of pseudoarthrosis	1,2,3: Complete recovery
8. Krödel et al. [35]	1997	1	55Y/F	Thoraco-lumbar scoliosis	T4-L4 Harrington distraction rod and compression system	6 years	Spastic Paraparesis	Removal of broken rod and decompression	T11-T12 compression by rod	Incomplete recovery
9. Papin et al. [36]	1999	1	15Y/F	AIS Thoraco-lumbar scoliosis	Pedicle screw fixation and correction	6 months	Right foot tremor, right ankle, right psoas and right triceps weakness with immediate post op severe abdominal and epigastric pain	Removal of right T8, T10 screws with return of SSEP	Convex T8, T10 screws encroaching the canal[> 4 mm] and compressing on right side	Complete recovery
10. Rittmeister et al. [37]	1999	1	29Y/F	AIS King Type 1	Hooks and rods T11-L3	8 years	Paraparesis with cauda equina syndrome	Complete removal of instrumentation with lumbar hook	Left-sided compression by laminar hook at L2	Complete recovery
11.Takahashi et al. [38]	2001	2	1. 59Y/F 2. 54Y/F	1. Degenerative thoraco-lumbar scoliosis 2. Degenerative lumbar scoliosis	1. T10 to L3 using stainless steel Cotrel-Dubousset instrumentation 2. T12 to L4 using stainless steel Cotrel-Dubousset instrumentation	1. 1.1 years 2. 4 years	1.Left L4-5 radiculopathy with claudication 2.Right L5 sciatica	1. Laminectomy L3 and L3 hook removal 2. Laminectomy of L4 and L5	1. Metallosis was observed at the hook–rod L3 junction with L2-3 pseudoarthrosis 2. Dark gray granulation tissue under the L4 lamina surrounding the right supralaminar hook of L4. metallosis around the hook–rod junction with pseudoarthrosis	1.Incomplete recovery 2.Complete recovery
12. Alanay et al. [39]	2003	1	6Y/F	T3-L2 progressive kyphosis	Fusion T2—L4 with lamino-transverse claw configurations at T3, T5, T7, and pedicle screw fixation of L2, L3, and L4	3 months	Paraparesis, dorsal column symptoms	Removal of T2 pedicle screws	Pull out of T2 pedicle screws may have caused cord compression as CSF leak detected intraoperatively	Complete recovery
13. Wolff et al. [40]	2005	1	26Y/F	T4-L1 Right thoracic curve with kyphosis and left L1-L4 curve	Fusion T4-L4 CD system with lumbar hooks	10 years	Paraplegia	Decompression of tumor and cord with removal of distal instrumentation	Intra dural extension of tumor along dural tear near L1 hook	Died due to tumor recurrence
14. Grisafi et al. [41]	2010	1	23Y/M	AIS	Anterior release with fusion followed by a posterior T3-L3 fusion with a hook and rod construct	8 years	Spastic paraparesis with sensory level at L1	Removal of all instrumentation	Severe canal compromise by a supra-laminar hook at T10 on the left, which was rotated inward. Bursal tissue around the hook sent for culture:growth coagulase-negative staphylococci	Complete recovery
15. Gardner et al. [42]	2012	1	38Y/M	T8-L3 Scoliosis	Posterior Harrington-Luque spinal fusion from T7—L3	22 years	Left lower limb paraparesis with radiculopathy and back pain	Pedicle screws above and below with removal of cyst and bone graft over pseudoarthrosis	Proximal pseudoarthrosis with degenerative cyst formation causing cord compression on left BX: degenerative cyst	Complete recovery
16. Vereijken et al. [43]	2013	4	1. 16Y/M 2. 14Y/M 3. 13Y/F 4. 15Y/F	1.T5-L1 AIS 2.T5-T10 AIS 3 T1-T5 T6-T12 T12-L4 AIS 4.T5-T11 T12-L3AIS	1.T5-L1 fusion hooks, rods 2.T4-T12 fusion with hooks, rods 3. T5-L1 fusion with hooks, rods 4.T3-L3 fusion with hooks and rods	1.2.4 years 2.6.6 years 3.1.7 years 4.5.7 years	1. Radicular pain in left flank 2.Cervico-thoracic pain, headache, nausea 3. Radicular pain left flank 4. Pain localized at proximal thoracic instrumentation and right leg hypoesthesia	1.Left rod and T5 hook removed 2. Left rod completely removed 3. T5 hook removed, transverse connector revised 4. T3 and T5 hook removed, proximal left and right rod removed	1.Left T5 hook compression with CSF leak 2. Left proximal hook T4 with CSF leak 3. Left proximal hook T5 4. T3,T5 hook with CSF leak	1.Complete recovery 2. Complete recovery 3. Complete recovery 4.Complete recovery
17. Mac-Thiong et al. [9]	2013	1	20Y/M	AIS	T4 to L2 posterior fusion	2 years	Progressive numbness below T4 dermatome and weakness in the left lower extremity Brown Sequard syndrome	Complete implant removal with T4 laminectomy and dural repair	Medial penetration of left T4 screw	Incomplete recovery
18. Obeid et al. [44]	2014	1	24Y/F	AIS	T2–L2 posterior arthrodesis	8 years	Spastic paraparesis	Implant removal	Intra-canal rod migration with complete laminar reconstitution T5-T10 from convex side. Cultures positive for *Propionibacterium acnes*	Complete recovery
19. Leroy et al. [45]	2016	1	19Y/M	Right thoracic scoliosis [Chiari 1, C6-C7 syrinx]	T2-L1 fusion with hooks, rods, and screws posteriorly	5 months	Right upper limb and right lower limb weakness with sensory ataxia left lower limb spastic signs with bladder involvement	Screw removal	Left T5 screw	Complete recovery (Spasticity disappeared)
20. Richman et al. [46]	2017	1	19Y/M	AIS	T4-LI fusion posterior	4 years	Low back pain and paresthesia on bilateral anterior thighs, flaccid paraparesis below knees and urinary retention	Decompression from T12 to L2 with removal of all instrumentation	Gritty yellow-black material tracking through the L1 foramen in the posterolateral epidural space causing thecal sac compression at T12-L2	Complete recovery
21. Ferrando et al. [47]	2017	3	1. 29Y/M 2. 21Y/M 3. 40Y/M	1.TL scoliosis 2. AIS TL scoliosis 3. TL scoliosis	1. T4-L4 TSRH hook-rod PSF 2.T5-T12 hook-rod construct 3. TSRH instrumentation T4-L2	1. 4 years 2. 5 years 3. 24 years	1. Paraparesis with conus medullaris syndrome L > R 2. Sensory ataxia no motor deficit 3. Sensory ataxia only	1. Complete removal of instrumentation 2.Implant removal 3. Implant removal	1.T10-T12 corrosion fatigue with metallosis with medial migration T3 hook 2. Spinal cord compression by laminar hooks at T10, T11, and T12. Distal portion of the hook-rod construct migrated medially 3. T11 hook found separated from the rod. Yellow-black material at the hook-rod junction	1. Complete recovery 2. Complete recovery 3. Incomplete recovery
22.Fernandes et al. [48]	2019	2	1. 16Y/F 2. 19Y/F	1.Downs, AIS right thoracic curve 2.Neuromuscular scoliosis West syndrome	1. T4–L4 posterior instrumentation 2. T2-to-pelvis fusion for a 100º neuromuscular scoliosis sublaminar wires through the dorsal spine and screws in the lumbosacral area	1. 2.3 years trauma car accident 2. 6 years	1.Spastic paraparesis, ataxia 2.Spastic paraparesis with bladder	1. Instrumentation removal 2. Implant removal	1.T5 pedicle hooks migrated into canal and covered by a layer of bone, progressive bony remodeling during migration 2. Left T2 and T3 canal migration of screw with pedicle osteolysis, with corrosion debris, culture Propionibacterium	1.Complete recovery 2. No recovery

## Case report

**Fig. 1:**
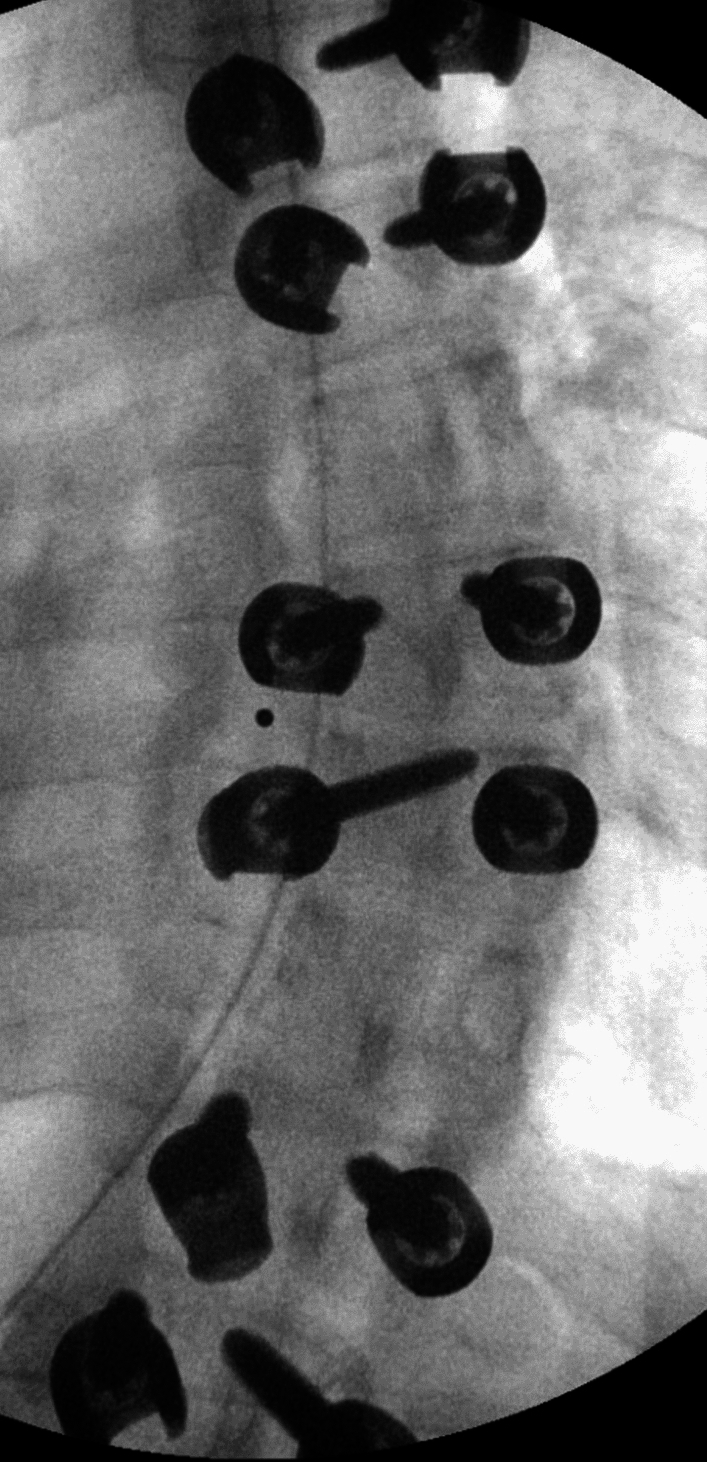
PA Fluorographic image taken intraoperatively (during the first surgery) showing the left T8 screw which is non-harmonious with the screws above and below

**Fig. 2:**
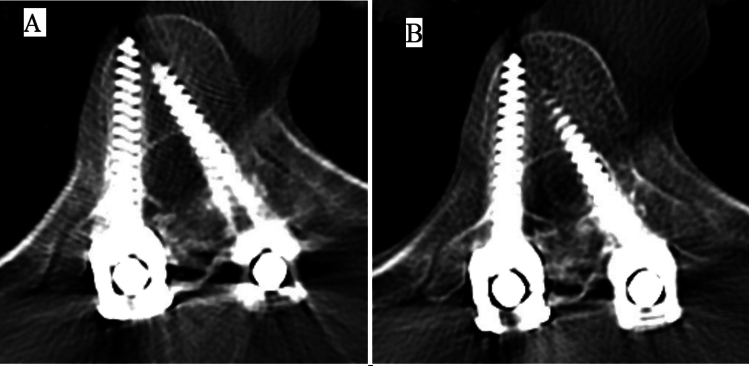
**A** Axial CT without contrast showing the left T8 screw breach into the spinal canal. **B** Axial CT myelogram at the level of T8 showing compression of thecal sac and cord by the medially misplaced screw

In 2015, a then 15-year-old patient presented to an outside orthopedic surgeon with adolescent idiopathic scoliosis. Radiology indicated a 49° main thoracic structural curve over a 45° lumbar curve. Patient underwent uneventful T4-L4 posterior spinal instrumentation and fusion; bilateral facetectomies were done at multiple thoracic levels and derotation maneuver was performed under fluoroscopic guidance (Figs. 1, 2). Post operatively the patient had no deficits and was discharged with an uncomplicated course. Neuromonitoring and intra-op CT was not done due to lack of availability and no follow-up CT scan was performed. The patient did well in immediate and long-term follow-up. Patient proceeded to work in a light manual labor job at a retail store.

**Fig. 3:**
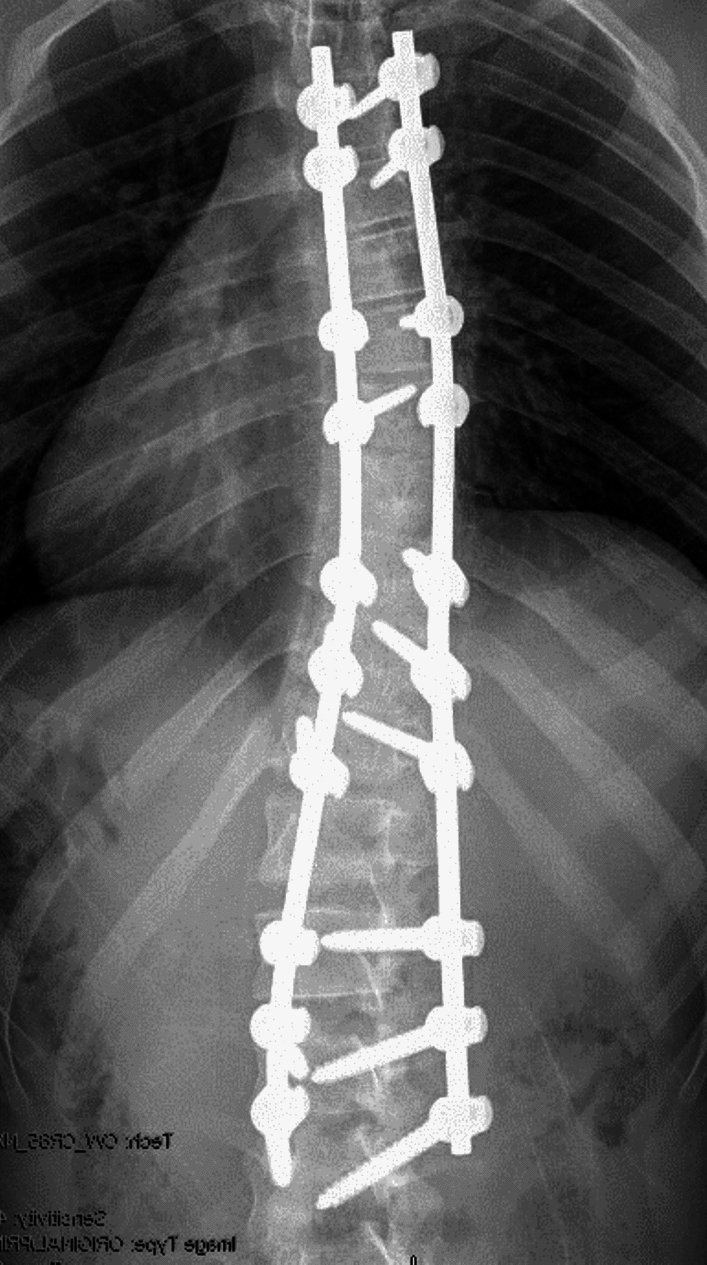
AP Fluorograph showing the post-operative correction after the first surgery

**Fig. 4:**
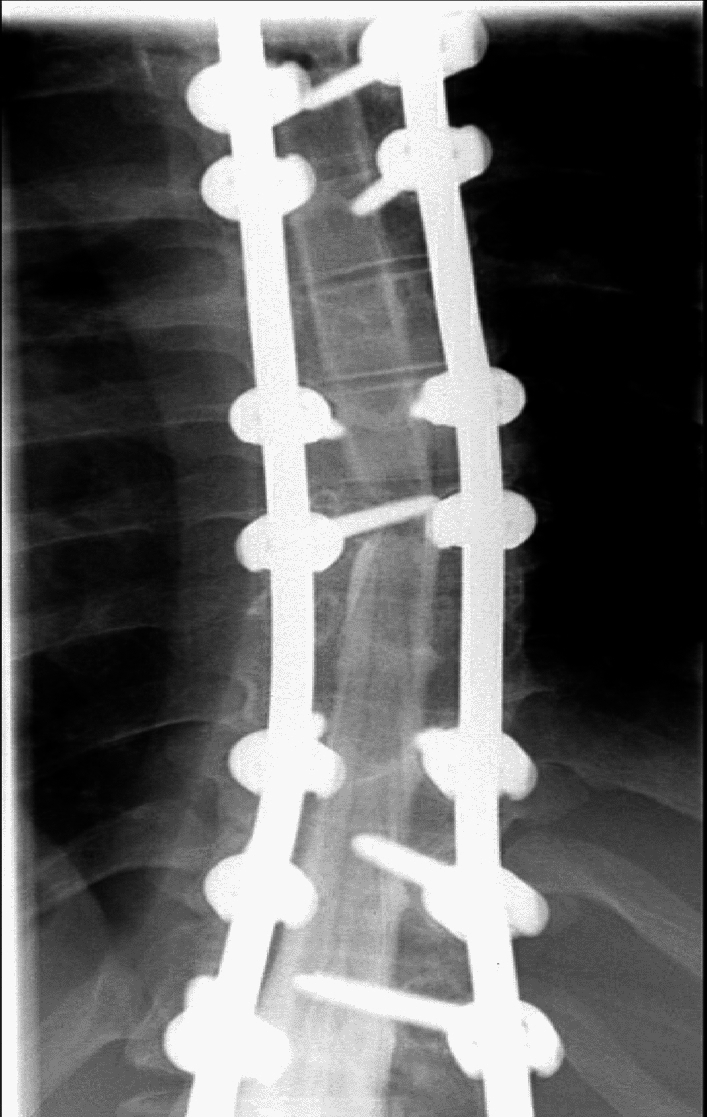
Fluorography with myelogram showing the left dura being impacted by the screw

Six years after surgery he presented to the emergency room in 2021 after sustaining a motor vehicle collision as a restrained passenger. He reported new onset of severe non-radiating back pain and bilateral lower extremity weakness immediately thereafter along with numbness and tingling in both lower limbs below the knee (L > R). There was no bladder and bowel incontinence, and his anal tone was normal. Motor power examination revealed a muscle power of left lower extremity 3/5 (all muscle groups), RLE 4 + /5 (all muscle groups) with normal upper limbs power of 5/5 after some initial BUE weakness. No Hoffmans or clonus was noted and his DTRs were 2 + . Sensory examination was intact. His MAP was increased to over 85 subsequently. CT brain, CT abdomen and other full trauma work up were negative. MRI of the cervical and lumbar spine were normal. MRI evaluation of the thoracic spine was limited in view of susceptibility artefact of T4-L4 Implants. There was no obvious posterior element disruption or hyperintensity. Patient then underwent a CT Thoracic spine with myelogram which revealed left T8 pedicle screw passing through the left lateral spinal canal and abutting the thecal sac/spinal cord with block of contrast material at that level (Figs. 3, 4). There was no evidence of pseudoarthrosis, spinal extradural hemorrhage or pedicle fracture.

**Fig. 5:**
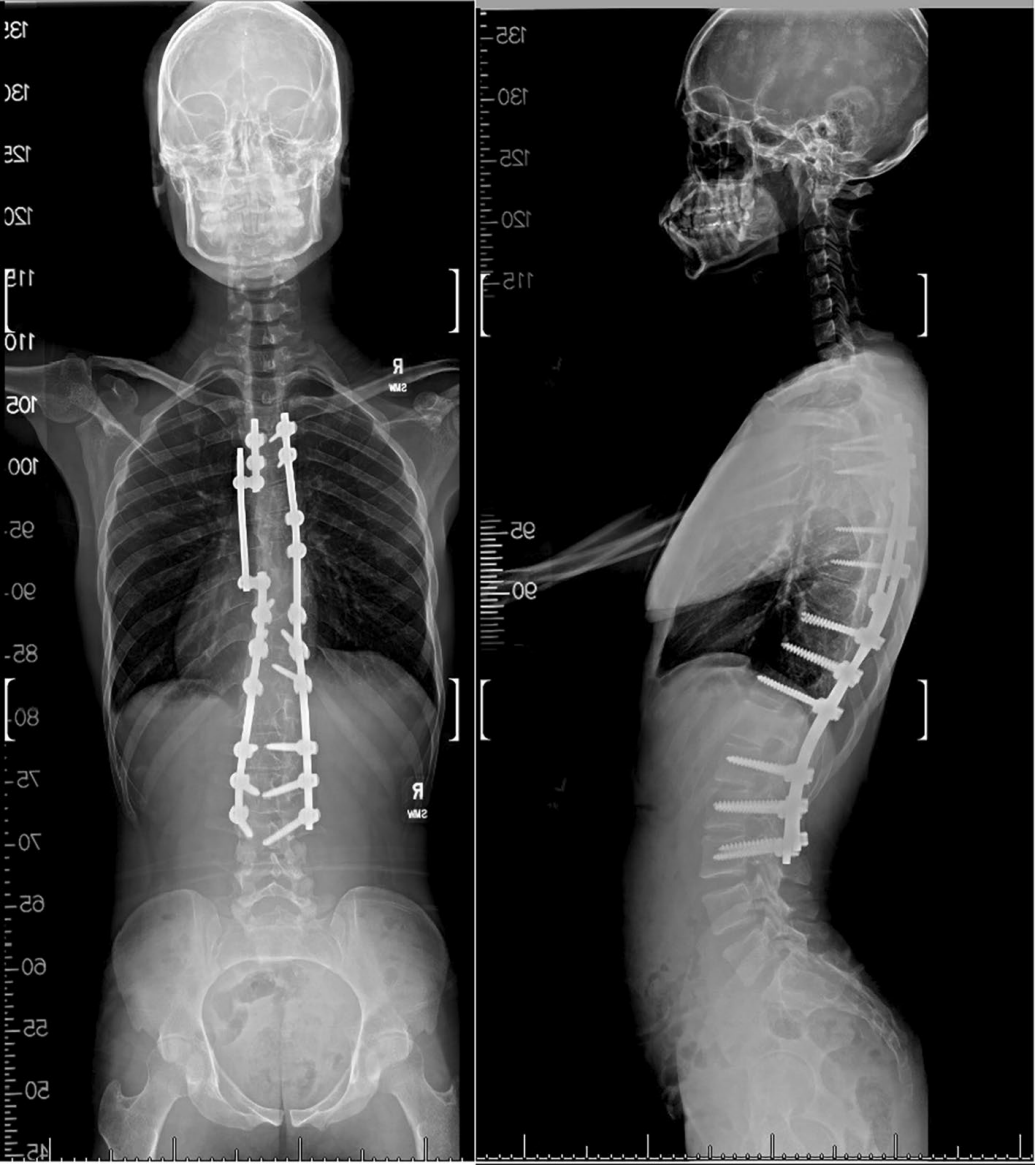
PA and lateral fluorograph showing post-operative correction, at 2 years follow-up showing the T6-T9 fixation. The T7 screw was removed for operative convenience

Patient then underwent T8 Left screw removal with T8 laminectomy and transpedicular decompression and pediculectomy from the left at T8 under neuromonitoring. A third rod was used to connect with an open type of lateral connector from T6 to T9 (Fig. 5). No CSF leak or EDH was seen, and the dura started pulsating after the T8 screw removal. There were no meaningful changes in neuromonitoring data either in SSEP or TcMEP during pre-positioning evaluation, post-positioning, or at the end of the case. There was side symmetry. He made significant improvement at the time of his discharge on POD3 with the left lower limb power improving to 4/5. Despite irregular interim follow-ups, the patient demonstrated a complete recovery of strength to 5/5 in both lower limbs and resolution of numbness and tingling after 2 years. However, some residual multifactorial pain remains. At the time of follow-up, his SRS-22 sub scores did remain low and he integrated with pain management status post-trauma; function—2.6/5, pain—2.2/5, self-image—3.0/5, mental health—2.0/5, and satisfaction/dissatisfaction with management—3.0/5.

## Discussion

### Case-specific considerations

Here we present a case of a delayed neurological deficit; (6 years) after the first AIS deformity surgery (T4-L4 PSF) under fluoroscopy guidance without neuro-navigation or neuromonitoring. The cause was determined to be the medially misplaced T8 pedicle screw which had been previously asymptomatic. The misplaced T8 left screw was present at the time of surgery itself as the intraoperative (first surgery) X-rays show the loss of harmonious progression of the screws above and below (Fig. 1). The patient had no motor or sensory deficit up until the trauma. After the trauma, CT did not reveal any pedicle fracture and the standing X-rays (compared to first post op) did not reveal any deformity progression, pull-out, or pseudoarthrosis. CT myelogram demonstrated that the screw was approximately 6.8 mm into the left canal and occupying 40% percent of the cross section of the canal at that level which suggests that the screw was intracanalicular [9]. The shape of the cord at the level of the screw compression was oblong in the CT myelogram as compared to the normal oval shape above and below, which indicates at-risk compression of the cord (Fig. 3B) [10, 11]. Intraoperatively there was no CSF leak, dural tear, extradural or intradural hemorrhage, infection or metallosis. Through retrospective subtraction of possibilities, it can be deduced that there was an unrecognized medial breach of the screw into the canal at the time of first surgery. This misplaced screw caused an at-risk stenosis of the spinal canal, which presented as a transient cord compression or potential contusion [10]. A normal neuromonitoring intraoperatively with an other functional neurological status ruled out a chronic compressive etiology. This, according to the authors’ knowledge is the first case report discussing the delayed symptomatic manifestation of an asymptomatic screw with motor vehicle collision as an inciting event.

Very few case reports in the literature have recognized this clinical problem of very late neurological sequelae developing months to years after a spinal deformity surgery. We have tabulated the list of all such case reports and classified the cause in each case. We chose to include cases which deteriorated at least 3 months after the first surgery thus completely excluding the immediate postop and early post op causes. 22 case reports describing 35 cases (1981 – 2019) were identified. There were 21 female cases and 13 male cases. In 1 case age/sex details were untraced. The shortest time to presentation of symptoms was 3 months and the longest was 25 years. The youngest case was aged 6 years and the oldest was aged 60 years at the time of presentation. The presentation was paraparesis in 14 cases (40%) with majority having spastic paraparesis 9 (25.7%), radiculopathy 11 (31.4%) cases, sensory ataxia in 4 cases (11.4%), bladder involvement in 3 (8.6%) monoparesis 2 (5.7%) cases, paraplegia (5.7%) in 2 cases, 1 (2.8%) cauda equina, Brown Sequard syndrome and conus medullaris syndrome. Hooks caused compression in 16 (45.7%) cases and were associated with sensory ataxia probably due to burrowing effect on the dura. Other causes of compression included bone hypertrophy in 5 cases (14.2%), rod in 5 cases (14.3%), leiomyosarcoma in 1 case and degenerative cyst in 1 case. Pseudoarthrosis was contributory in 7 (20%) cases and wire breakage and infection were associated in 2 (5.7%) cases each. CSF leak in 3 cases (8.6%), chronic dural tear in 2 cases (5.7%), metallosis 6 cases (17.1%) were also observed intraoperatively.

In 5 cases (14.3%) the screws caused compression and neurodeficit, but in none of these cases, a high-speed MVC trauma preceded the neurological decline. 25 cases achieved complete recovery post removal of the offending hardware (71.4%), 5 patients had partial recovery (14.3%), one patient died of tumor recurrence. Growth and deformity progression may lead to implant migration and the spine “growing” around the implants and neurological compromise as seen in the case of Fernandes et al.

### Specific challenges in a concavity-based pedicle screw

The incidence of pedicle screw misplacement among thoracic pedicles in spinal deformities ranges from 3 to 25%, with screw-related neurological complications in 0–0.9% [4]. The frequency of medial perforation of screws placed in thoracic pedicles is relatively higher (1.7–26%) and especially occurs at T3-T8 levels [12]. Thoracic concavity-based pedicle screws are potentially more at risk due to the small pedicle diameter and less space between the cord and medial pedicle (1 mm on concave side). In severe deformities, the concave side pedicles are thinner, more sclerotic, and dysplastic with the cord in direct contact with the medial wall of the pedicle. [13]. In other presentations, thoracic medial perforations are tolerable up to 4 mm (2 mm in the epidural space and 2 mm in the subarachnoid space) [14]. Kim et al. defined the safe zone of medial perforation in scoliotic patient as a ‘‘definite’’ safe zone (2 mm), a ‘‘probable’’ safe zone (2–4 mm), and a ‘‘questionable’’ safe zone (4–8 mm) [4].The spinal cord is tethered in large stiff curves on the concave side, even during correction and a slightly misplaced screw may have a significant consequence in large and stiff curves. These literature findings suggest that there is minimal safety zone on the concave side. Conversely the plasticity of the immature cortical bone in young patients can be protective and allows significant deformation up to 200% of the pedicle wall [15, 16]. This may also have the potential to be space limiting. Indeed, in our patient, this was a concavity-based screw.

## Prophylactic considerations

### Verifying placement accuracy

Various means to identify screw misplacement have been described in the literature. Radiological parameters suggested by Kim and Lenke et al. [4] to detect medial and lateral breach are: (1) Violation of the harmonious segmental change of the tips of screws with reference to the vertebral rotation using the posterior upper spinolaminar junction (medial or lateral out) (2) No crossing of the medial pedicle wall by the tip of the pedicle screw inserted with reference to the vertebral rotation using the posterior upper spinolaminar junction (lateral out); and (3) Violation of the imaginary midline of the vertebral body using the posterior upper spinolaminar junction by the position of the tip of the inserted pedicle screw (medial out).

This can also be evaluated with intra-operative or even immediately post-operative CT to illustrate the safe location. The most commonly used postoperative imaging technique is CT scan [17]. Low-radiation dose CBCT is an alternative with 20 times less radiation [18]. Any new onset of neurological symptoms should prompt the surgeon to thoroughly investigate the cause, with CT at the earliest. Another more esoteric method involves fluoroscopic contrast dye injection in the pedicle tract before screw placement and visualizing the leak [19].

Neuromonitoring is critical. Intraoperatively a breach can be evident by the changes in neuromonitoring. tEMG [threshold 10–12 mA, pulse duration 300 μsec] of pedicle screw works best for detecting medial and foraminal screw misplacement [20]. Also a disconcordance in relative number can also be used. It is important that all this information be integrated along with intra-operative imaging. Enabling technology may also have a role in certain settings.

### Medially misplaced screws whether to remove or not?

The decision-making for an asymptomatic screw removal is formidable after surgery. Therefore, as stated above, it is important to recognize this during the intra-operative period. Pedicle screw misplaced medially greater than 2 mm, especially 4 mm (scoliosis patients) should have a potential low threshold for removal in the early phase, even without neurological symptoms [12, 21]. Logically the clinical manifestation of a breach and its risk should determine its treatment more than the degree of breach. The reported rate of unplanned return to the operating room for a misplaced screw is 0.17–4.3% [22–25] and redirection may compromise biomechanical integrity, with a 28% loss of pull-out strength [24]. Each surgeon must make the decision to remove instrumentation on a case-by-case basis weighing the patient specific risk and benefits.

Our patient underwent surgery for AIS and it is unknown to us if he ever had any objective symptoms prior to our trauma. He returned to what he describes as normal activity. Minimal evidence or guidelines exist on the recommended timeline to return to full range of motion after adult spinal deformity surgery. Integrity of instrumentation is an important part of that calculus. Theologis et al. [8] formulated a perioperative spine survey by a study group within AO spine (high-volume spine surgeons); which recommended return to unrestricted range of motion within 3 months of ASD surgery, to non-contact sports within 4 months and waiting at least 4 months after surgery to return to contact sports. Some of the limitations of this study were, low response rate of the (~ 5%), which may have introduced selection bias. Also, the study did not provide more detailed information on patient factors (i.e., age, DEXA scores, fitness levels, frailty, psychiatric profiles) and specific surgical details (i.e., exact levels of fusion, instrumentation/rod materials, interbody support, cement augmentation, alignment) and a lack of a clear distinction between contact sports vs collision sports.

Barile et al. [26] recommended that AIS patients can safely return to sports between 6 and 18 months after surgery; even to extreme sports; although (long fusions and distal fusions), the loss of mobility could make it difficult for them to attain preoperative level of competence. Hence, the decision regarding return to activity after spine deformity surgery should be surgeon specific and based on his experience and intraoperative findings.

## Limitations

This case report and data inherently entail highly individualized outcome predictability. A limitation arises from the lack of access to full preoperative imaging due to surgery conducted outside our institution, which limits the completeness of the clinical narrative. While intraoperative images from the initial surgery depict a loss of harmonious screw progression, our use of SRS-22 scores for patient-reported outcomes is constrained by the absence of baseline calculations. Additionally, the patient's initial presentation was due to a motor vehicle collision rather than specifically for scoliosis, which adds complexity to the interpretation of the outcomes.

## Conclusion

A rare case of a delayed neurological deficit following spine deformity correction in a young boy presented with paraparesis after 6 years, following motor vehicle high-speed collision. Medial pedicle screw misplacement has a variable, but low incidence and the complications can be clinically significant. What can be controlled is the accurate screw placement and detection of the misplacement using various adjuncts. An asymptomatic misplacement needs to be evaluated on a case-by-case basis relying on clinical features, location, and degree of breach [27]. The return to activity decision should be patient and surgeon specific and expert recommendations may be tailored with regional practice patterns.

## Data Availability

The datasets generated and/or analyzed during the current study are available from the corresponding author upon reasonable request. Data will be sent through an email to the requesting party. To request data access, message the corresponding author, Dr. Sudhir Suggala (sudhirsuggala@gmail.com).

## References

[1] Boucher HH (1959) A method of spinal fusion. The J of Bone & Jt Surg British 41(2):248–259 10.1302/0301-620X.41B2.248 13641310

[2] King D (1948) Internal fixation for lumbosacral fusion. J Bone Jt Surg Am 30(3):560–578 18109577

[3] Suk SI, Kim WJ, Lee SM, Kim JH, Chung ER (2001) Thoracic pedicle screw fixation in spinal deformities: are they really safe? Spine 26(18):2049–2057. 10.1097/00007632-200109150-00022 11547207 10.1097/00007632-200109150-00022

[4] Kim YJ, Lenke LG, Cheh G, Riew KD (2005) Evaluation of pedicle screw placement in the deformed spine using intraoperative plain radiographs: a comparison with computerized tomography. Spine 30(18):2084–2088 16166900 10.1097/01.brs.0000178818.92105.ec

[5] Keyoung HM, Kanter AS, Mummaneni PV (2008) Delayed-onset neurological deficit following correction of severe thoracic kyphotic deformity: case report. J Neurosurg Spine 8(1):74–79. 10.3171/SPI-08/01/074 18173350 10.3171/SPI-08/01/074

[6] Leong JJH, Curtis M, Carter E, Cowan J, Lehovsky J (2016) Risk of neurological injuries in spinal deformity surgery. Spine 41(12):1022–1027. 10.1097/BRS.0000000000001366 26679891 10.1097/BRS.0000000000001366

[7] Qiao J, Xiao L, Zhu Z et al (2018) Delayed postoperative neurologic deficit after spine deformity surgery: analysis of 5377 cases at 1 institution. World Neurosurg 111:e160–e164. 10.1016/j.wneu.2017.12.010 29253692 10.1016/j.wneu.2017.12.010

[8] Theologis AA, Cummins DD, Kato S et al (2023) Activity and sports resumption after long segment fusions to the pelvis for adult spinal deformity: survey results of AO spine members. Spine Deform 11(6):1485–1493. 10.1007/s43390-023-00734-6 37462878 10.1007/s43390-023-00734-6 PMC10587314

[9] Mac-Thiong JM, Parent S, Poitras B, Joncas J, Hubert L (2013) Neurological outcome and management of pedicle screws misplaced totally within the spinal canal. Spine 38(3):229–237. 10.1097/BRS.0b013e31826980a9 22814305 10.1097/BRS.0b013e31826980a9

[10] Boese CK, Lechler P (2013) Spinal cord injury without radiologic abnormalities in adults: a systematic review. J Trauma Acute Care Surg 75(2):320–330. 10.1097/TA.0b013e31829243c9 23702634 10.1097/TA.0b013e31829243c9

[11] Asan Z (2018) Spinal concussion in adults: transient neuropraxia of spinal cord exposed to vertical forces. World Neurosurg 114:e1284–e1289. 10.1016/j.wneu.2018.03.198 29626691 10.1016/j.wneu.2018.03.198

[12] Sugawara R, Tsuji T, Saito T, Nohara A, Kawakami K, Kawakami N (2015) Medially misplaced pedicle screws in patients without neurological deficits following scoliosis surgery: to observe or to remove? Eur Spine J 24(7):1450–1456. 10.1007/s00586-015-3860-y 25749727 10.1007/s00586-015-3860-y

[13] Liljenqvist UR, Halm HFH, Link TM (1997) pedicle screw instrumentation of the thoracic spine in idiopathic scoliosis. Spine 22(19):2239–2245. 10.1097/00007632-199710010-00008 9346144 10.1097/00007632-199710010-00008

[14] Gertzbein SD, Robbins SE (1990) Accuracy of pedicular screw placement in vivo. Spine 15(1):11–14. 10.1097/00007632-199001000-00004 2326693 10.1097/00007632-199001000-00004

[15] Yazici M, Pekmezci M, Cil A, Alanay A, Acaroglu E, Oner FC (2006) The effect of pedicle expansion on pedicle morphology and biomechanical stability in the immature porcine spine. Spine 31(22):E826–E829. 10.1097/01.brs.0000240759.06855.e6 17047529 10.1097/01.brs.0000240759.06855.e6

[16] Cho SK, Skovrlj B, Lu Y, Caridi JM, Lenke LG (2014) The effect of increasing pedicle screw size on thoracic spinal canal dimensions: an anatomic study. Spine 39(20):E1195–E1200. 10.1097/BRS.0000000000000514 25010092 10.1097/BRS.0000000000000514

[17] Aoude AA, Fortin M, Figueiredo R, Jarzem P, Ouellet J, Weber MH (2015) Methods to determine pedicle screw placement accuracy in spine surgery: a systematic review. Eur Spine J 24(5):990–1004. 10.1007/s00586-015-3853-x 25749690 10.1007/s00586-015-3853-x

[18] Burström G, Cewe P, Charalampidis A et al (2021) Intraoperative cone beam computed tomography is as reliable as conventional computed tomography for identification of pedicle screw breach in thoracolumbar spine surgery. Eur Radiol 31(4):2349–2356. 10.1007/s00330-020-07315-5 33006659 10.1007/s00330-020-07315-5 PMC7979653

[19] Jeon CH, Chung NS (2014) A simple intraoperative method for assessment of pedicle screw trajectory using contrast medium injection. J Spinal Disord Tech 27(1):E14–E19. 10.1097/BSD.0b013e3182886fd7 23429313 10.1097/BSD.0b013e3182886fd7

[20] Mikula AL, Williams SK, Anderson PA (2016) The use of intraoperative triggered electromyography to detect misplaced pedicle screws: a systematic review and meta-analysis. J Neurosurg Spine 24(4):624–638. 10.3171/2015.6.SPINE141323 26654343 10.3171/2015.6.SPINE141323

[21] Floccari LV, Larson AN, Crawford CH et al (2018) Which malpositioned pedicle screws should be revised? J Pediatr Orthop 38(2):110–115. 10.1097/BPO.0000000000000753 27078232 10.1097/BPO.0000000000000753

[22] Odate S, Fujibayashi S, Otsuki B et al (2022) Reoperation for misplaced pedicle screws: a multicenter retrospective study. Spine 47(21):1525–1531. 10.1097/BRS.0000000000004398 35797598 10.1097/BRS.0000000000004398

[23] Di Silvestre M, Parisini P, Lolli F, Bakaloudis G (2007) Complications of thoracic pedicle screws in scoliosis treatment. Spine 32(15):1655–1661. 10.1097/BRS.0b013e318074d604 17621214 10.1097/BRS.0b013e318074d604

[24] Amaral TD, Hasan S, Galina J, Sarwahi V (2021) Screw malposition: are there long-term repercussions to malposition of pedicle screws? J Pediatr Orthop 41(Suppl 1):S80–S86. 10.1097/BPO.0000000000001828 34096543 10.1097/BPO.0000000000001828

[25] Sankey EW, Mehta VA, Wang TY et al (2020) The medicolegal impact of misplaced pedicle and lateral mass screws on spine surgery in the United States. Neurosurg Focus 49(5):E20. 10.3171/2020.8.FOCUS20600 33130620 10.3171/2020.8.FOCUS20600

[26] Barile F, Ruffilli A, Manzetti M et al (2021) Resumption of sport after spinal fusion for adolescent idiopathic scoliosis: a review of the current literature. Spine Deform 9(5):1247–1251. 10.1007/s43390-021-00330-6 33759111 10.1007/s43390-021-00330-6 PMC8363544

[27] Aoude A, Ghadakzadeh S, Alhamzah H et al (2018) Postoperative assessment of pedicle screws and management of breaches: a survey among canadian spine surgeons and a new scoring system. Asian Spine J 12(1):37–46. 10.4184/asj.2018.12.1.37 29503680 10.4184/asj.2018.12.1.37 PMC5821931

[28] Eismont FJ, Simeone FA (1981) Bone overgrowth (hypertrophy) as a cause of late paraparesis after scoliosis fusion. A case report. J Bone Jt Surg Am 63(6):1016–1019 7240319

[29] Court-Brown CM, McMaster MJ (1982) Pseudarthrosis: a late cause of paraparesis after scoliosis surgery. A case report. J Bone Jt Surg Am 64(8):1246–1248 7130237

[30] Bernard TN Jr, Johnston CE 2nd, Roberts JM, Burke SW (1983) Late complications due to wire breakage in segmental spinal instrumentation. Report of two cases. J Bone Jt Surg Am 65(9):1339–1345 6654946

[31] Roy DR, Huntington CF, MacEwen GD (1984) Pseudarthrosis resulting in complete paraplegia fifteen years after spinal fusion. Arch Orthop Trauma Surg 102(4):213–215. 10.1007/BF00436131 6712420 10.1007/BF00436131

[32] Kornberg M, Herndon WA, Rechtine GR (1985) Lumbar nerve root compression at the site of hook insertion: late complication of Harrington rod instrumentation for scoliosis. Spine 10(9):853–855. 10.1097/00007632-198511000-00015 2935952 10.1097/00007632-198511000-00015

[33] Hales DD, Dawson EG, Delamarter R (1989) Late neurological complications of Harrington-rod instrumentation. J Bone Jt Surg Am 71(7):1053–1057 2668293

[34] Savini R, Di Silvestre M, Gargiulo G (1990) Late paraparesis due to pseudarthrosis after posterior spinal fusion. J Spinal Disord 3(4):427–432 2134461

[35] Krodel A, Rehmet JC, Hamburger C (1997) Spinal cord compression caused by the rod of a Harrington instrumentation device: a late complication in scoliosis surgery. Eur Spine J 6(3):208–210. 10.1007/BF01301439 9258642 10.1007/BF01301439 PMC3454621

[36] Papin P, Arlet V, Marchesi D, Rosenblatt B, Aebi M (1999) Unusual presentation of spinal cord compression related to misplaced pedicle screws in thoracic scoliosis. Eur Spine J 8(2):156–159. 10.1007/s005860050147 10333156 10.1007/s005860050147 PMC3611145

[37] Rittmeister M, Leyendecker K, Kurth A, Schmitt E (1999) Cauda equina compression due to a laminar hook: a late complication of posterior instrumentation in scoliosis surgery. Eur Spine J 8(5):417–420. 10.1007/s005860050197 10552327 10.1007/s005860050197 PMC3611190

[38] Takahashi S, Delécrin J, Passuti N (2001) Intraspinal metallosis causing delayed neurologic symptoms after spinal instrumentation surgery. Spine 26(13):1495–1498. 10.1097/00007632-200107010-00024 11458158 10.1097/00007632-200107010-00024

[39] Alanay A, Cil A, Acaroglu E et al (2003) Late spinal cord compression caused by pulled-out thoracic pedicle screws: a case report. Spine 28(24):E506–E510. 10.1097/01.BRS.0000099389.96750.6D 14673376 10.1097/01.BRS.0000099389.96750.6D

[40] Vialle R, Wolff S, David P, Lepeintre JF, Hautefort P, Tadie M (2005) Late Paraplegia after scoliosis treatment: an uncommon diagnosis. J Spinal Disord Tech 18(6):531–534. 10.1097/01.bsd.0000132285.19961.30 16306845 10.1097/01.bsd.0000132285.19961.30

[41] Grisafi FN, Emery SE (2010) Migration of laminar hook causing thoracic myelopathy eight years post scoliosis surgery: a case report. Spine 35(6):E228–E230. 10.1097/BRS.0b013e3181bf20a4 20195204 10.1097/BRS.0b013e3181bf20a4

[42] Gardner A (2012) Paraparesis caused by a cyst in the spinal canal from a pseudarthrosis 22 years following Harrington rod procedure for scoliosis: a case report. J Med Case Reports 6(1):337. 10.1186/1752-1947-6-337 10.1186/1752-1947-6-337 PMC3470989 23034101

[43] Vereijken IMP, De Kleuver M (2013) Late proximal pedicle hook migration into spinal canal after posterior correction surgery of scoliosis causing neurologic deficit: “proximal junctional scoliosis”? Case series and a review of the literature. Spine Deform 1(3):229–236. 10.1016/j.jspd.2013.04.001 27927298 10.1016/j.jspd.2013.04.001

[44] Obeid I, Vital JM, Aurouer N et al (2016) Intraspinal canal rod migration causing late-onset paraparesis 8 years after scoliosis surgery. Eur Spine J 25(7):2097–2101. 10.1007/s00586-014-3367-y 24903395 10.1007/s00586-014-3367-y

[45] Leroy A, Kabbaj R, Dubory A, Bachy M, Vermersch AI, Vialle R (2016) The Indian Basket Trick: a case of delayed paraplegia with complete recovery, caused by misplaced thoracic pedicle screw. Springerplus 5(1):944. 10.1186/s40064-016-2334-y 27386388 10.1186/s40064-016-2334-y PMC4929096

[46] Richman SH, Razzano AJ, Morscher MA, Riley PM (2017) Metallosis presenting as a progressive neurologic deficit four years after a posterior spinal fusion for adolescent idiopathic scoliosis: a case report. Spine 42(1):E56–E59. 10.1097/BRS.0000000000001685 27172284 10.1097/BRS.0000000000001685

[47] Ferrando A, Bas P, Bas T (2017) Late neurological complications due to laminar hook compression in idiopathic scoliosis surgery. Spinal Cord Ser Cases 3(1):17081. 10.1038/s41394-017-0009-8 29423287 10.1038/s41394-017-0009-8 PMC5798914

[48] Fernandes P, Soares Do Brito J, Flores I, Monteiro J (2019) Impact of surgery on the quality of life of adolescent idiopathic scoliosis. Iowa Orthop J 9(2):66–72 PMC7047296 32577110

